# The influence of sighing respirations on infant lung function measured using multiple breath washout gas mixing techniques

**DOI:** 10.14814/phy2.12347

**Published:** 2015-04-06

**Authors:** Damjan Vukcevic, John B Carlin, Louise King, Graham L Hall, Anne-Louise Ponsonby, Peter D Sly, Peter Vuillermin, Sarath Ranganathan

**Affiliations:** 1 Data Science, Murdoch Childrens Research InstituteParkville, Victoria, Australia; 2 Department of Mathematics and Statistics, Faculty of Science, University of MelbourneParkville, Victoria, Australia; 3 Department of Paediatrics, Faculty of Medicine, Dentistry and Health Sciences, University of MelbourneParkville, Victoria, Australia; 4 Melbourne School of Population and Global Health, Faculty of Medicine, Dentistry and Health Sciences, University of MelbourneParkville, Victoria, Australia; 5 Child Health Research Unit, Barwon HealthGeelong, Victoria, Australia; 6 Infection and Immunity, Murdoch Childrens Research InstituteParkville, Victoria, Australia; 7 Department of Respiratory Medicine, Royal Children's HospitalParkville, Victoria, Australia; 8 Paediatric Respiratory Physiology, Telethon Kids Institute, University of Western AustraliaPerth, Western Australia, Australia; 9 Population Health, Murdoch Childrens Research InstituteParkville, Victoria, Australia; 10 Queensland Children's Medical Research Institute, The University of QueenslandBrisbane, Queensland, Australia; 11 School of Medicine, Faculty of Health, Deakin UniversityGeelong, Victoria, Australia

**Keywords:** Infants, lung function, multiple breath washout, sighing respirations

## Abstract

There is substantial interest in studying lung function in infants, to better understand the early life origins of chronic lung diseases such as asthma. Multiple breath washout (MBW) is a technique for measuring lung function that has been adapted for use in infants. Respiratory sighs occur frequently in young infants during natural sleep, and in accordance with current MBW guidelines, result in exclusion of data from a substantial proportion of testing cycles. We assessed how sighs during MBW influenced the measurements obtained using data from 767 tests conducted on 246 infants (50% male; mean age 43 days) as part of a large cohort study. Sighs occurred in 119 (15%) tests. Sighs during the main part of the wash-in phase (before the last 5 breaths) were not associated with differences in standard MBW measurements compared with tests without sighs. In contrast, sighs that occurred during the washout were associated with a small but discernible increase in magnitude and variability. For example, the mean lung clearance index increased by 0.36 (95% CI: 0.11–0.62) and variance increased by a multiplicative factor of 2 (95% CI: 1.6–2.5). The results suggest it is reasonable to include MBW data from testing cycles where a sigh occurs during the wash-in phase, but not during washout, of MBW. By recovering data that would otherwise have been excluded, we estimate a boost of about 10% to the final number of acceptable tests and 6% to the number of individuals successfully tested.

## Introduction

Epidemiological studies suggest that the risk of developing chronic lung disease (CLD), such as asthma and COPD, may be altered by perinatal (Svanes et al. 2004; Devereux et al. 2006; Lannero et al. 2006; Bush 2008; Hylkema and Blacquiere 2009; Bekkers et al. 2012) and early postnatal exposures (Kusel et al. 2007; Jackson et al. 2008). However, the relevant developmental windows and underlying mechanisms, including the extent to which specific factors influence risk of CLD, are poorly understood (Krauss-Etschmann et al. 2013). In this context there is substantial interest in techniques for measuring lung function during early infancy. Multiple breath washout (MBW) testing assesses the efficiency of inert gas clearance from the lung, and hence, ventilation efficiency (Fowler 1949; Robertson et al. 1950).

MBW is a sensitive measure of obstructive lung disease (Horsley et al. 2008; Aurora et al. 2011); and unlike conventional spirometry, MBW may be performed in young infants, as well as older children and adults (Robinson et al. 2013). Furthermore, unlike many other techniques for measuring lung function in infants, MBW may be conducted during natural, unsedated sleep (Fuchs et al. 2011). It is, however, time consuming and challenging to obtain adequate MBW measurements in infants during natural sleep.

Current guidelines recommend the exclusion of measurements during which sigh breaths or yawns occur within the period 10 breaths prior to achieving equilibration (the wash-in phase) or during the first 10 breaths of the washout (Robinson et al. 2013). Infants sigh frequently during sleep (Alvarez et al. 1993) so adoption of this guideline renders a relatively large proportion of MBW testing cycles unreportable. There are currently few data to formally evaluate the recommendation to exclude testing cycles containing sighs. The aim of this study was to determine the influence of sighs on the key measures of lung function derived from MBW testing in the wash-in and washout phases of testing during natural, unsedated sleep in early infancy.

## Methods

### Participants

MBW measurements were conducted among participants in the Barwon Infant Study (BIS). BIS is a population-derived birth cohort study (*n* = 1074) with antenatal recruitment, conducted in the south-east of Australia, designed to investigate the early life origins of a range of noncommunicable diseases. Participants were invited to undergo MBW testing at 1 month of age. Among the 982 infants to complete the 1 month review, 654 (67%) consented to MBW testing. MBW was attempted among 570/654 (87%) consented infants who were free from respiratory illness and fell asleep during the course of the 2 hour review. Acceptable and reproducible MBW measurements were obtained in 318 infants (56% (318/570) of those tested; 30% (318/1074) of the complete cohort).

For the analyses reported here, data were taken from tests conducted within the first 17 months of the study (Feb 2011 to Jun 2012, inclusive), and the standard acceptability and reproducibility criteria were modified to suit the research question (see below). A total of 246 infants had tests that met these constraints.

### MBW testing technique and protocol

Ventilation inhomogeneity was measured in infants between 4 to 12 weeks of age during natural sleep by MBW using sulfur hexafluoride (SF_6_) and an ultrasonic flowmeter (ExhalyzerD, Ecomedics, Duernten, Switzerland). Testing was performed and analyzed as reported by our group previously (Schibler et al. 2002; Latzin et al. 2007) in accordance with current guidelines (Robinson et al. 2013). Participants were required to be free from respiratory tract illness for at least 3 weeks prior to testing. The wash-in gas comprised 4% SF_6_, 21% oxygen and balance nitrogen. Case temperature and relative humidity in the ExhalyzerD system were set at constant values of 25°C and 20% respectively across each test. A size 1 Laerdal face mask was used (calculated dead space value of 0.012 L).

Tests were considered technically acceptable if there was a stable breathing pattern throughout the test (other than the presence of up to a single sigh), with no other artifacts present, such as sucking, snoring, mask leaks, or breath holds.

A sigh was defined as a marked increase (at least double) in tidal volume with no other artefacts present. When a sigh was present, it was categorized depending on the temporal position of the sigh into one of the following:

- Sigh during wash-in, before the last 5 breaths (‘washin-pre’)
- Sigh during wash-in, within the last 5 breaths (‘washin-post’)
- Sigh during washout, prior to and including the 10 breaths after reaching 1/40th of the starting tracer gas concentration (‘washout-pre’)
- Sigh during washout, in any breath in the period after washout-pre (‘washout-post’).

**Figure 1:**
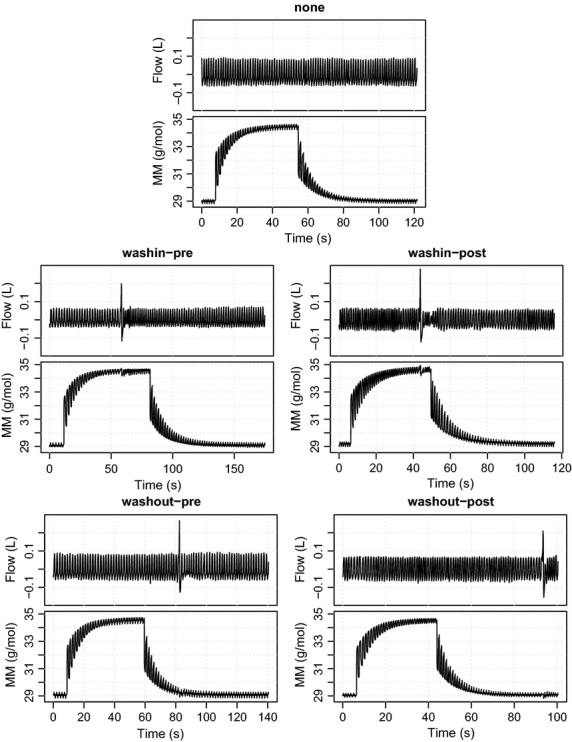
Raw data series from MBW tests, showing one example from each of the five scenarios (none, washin-pre, washin-post, washout-pre, washout-post). For each scenario, high-frequency series of the gas flow (Flow) and gas density (MM) measurements are shown.

Each test therefore corresponds to one of five different *scenarios*: either a sigh in one of the four positions described above, or no sigh (‘none’). Figure1 shows data series obtained from a single lung function test from each of these five scenarios. We defined the above scenarios before the publication of the current guidelines (Robinson et al. 2013). There is no direct analog of the *critical period* as defined in the guidelines. Notably, when taken together with our recommendations (see Discussion), our threshold for differentiating sighs during wash-in is more liberal (allowing sighs up until the last 5 breaths, rather than the last 10 breaths), while that for the washout is more stringent (excluding sighs even beyond the first 10 breaths of the washout).

Each test was analyzed and reported after correcting for BTPS (body temperature and pressure saturated).

Five outcome variables were calculated for each test:

- Functional residual capacity (FRC)
- Cumulative expired gas volume (CEV)
- Lung clearance index (LCI)
- Moment ratio M_1_/M_0_ (MR1)
- Moment ratio M_2_/M_0_ (MR2).

These were defined according to standard protocols (Robinson et al. 2013) and were calculated using the WBreath software package (version 3, 19, 6, 0, ndd Medizintechnik AG, Zurich, Switzerland), which was provided with the measurement equipment.

Ordinarily, tests would also be evaluated against standard reproducibility criteria. These assess whether a set of measurements for the same individual are similar enough to be considered suitable for further analyses. Typically, this leads to the exclusion of highly variable tests. Since our aim here is precisely to study the variability in outcome measurements, we did *not* exclude any tests in this manner because it would bias the observed variability. However, we did apply such criteria to assess the benefit of including certain tests with sighs into a study, by evaluating the resulting increase in sample size, in terms of the number of tests and number of individuals that would be available for analysis. We used the following criteria, which are consistent with the ERS/ATS consensus statement (Robinson et al. 2013):

- If only 1 test was available for an individual, it was declared as NOT reproducible.
- If exactly 2 tests were available, we declared them as reproducible if the smaller functional residual capacity (FRC) value was within 10% of the larger FRC value, otherwise both were declared as NOT reproducible.
- If at least 3 tests were available, we first calculated the median FRC values across all tests. We then determined how many tests had their FRC values within 25% of this median value. If there were at least three such tests, we declared them as reproducible and all the ones outside the 25% window as NOT reproducible. If there were fewer than three tests within the 25% window, then *all* tests were declared as NOT reproducible.

### Statistical methods

A key feature of our data were repeated measurements, with a varying number of replicates across individuals. We used a linear mixed-effects model to explicitly account for this structure in our data. This is not possible with, for example, a standard Bland-Altman-style comparison analysis.

Our model allows a different mean and variance for each sigh scenario, while allowing for variation between individuals and any interaction between scenarios and individuals. See the Appendix for full details of the model. The analysis was carried out using the R software environment (R Core Team 2014) with the nlme package (Pinheiro et al. 2014).

We fitted the model separately to each of the five outcome variables (LCI, CEV, FRC, MR1, MR2). We checked the model fit by inspecting the standardized residuals across each of the sighs categories.

A small number of tests had unusually large values for some outcome variables (see Results for details) and had a noticeable influence on the model fit. We chose to exclude these unusual values from the analysis in order to obtain a model that adequately describes the vast majority of the data. In practice, any unusually extreme values of the outcome variables would also likely be excluded, or at least closely scrutinized. In fact, all of these unusual values end up being excluded after the application of the reproducibility criteria (described above).

To assess agreement between scenarios, we calculated 95% limits of agreement based on comparing two types of hypothetical measurement done on the same individual:

- The mean of three replicates from tests without sighs.
- The mean of three replicates from two tests without a sigh and one test with a sigh (the sigh can come from any of the four scenarios with a sigh).

This is intended to mimic the way these measurements would be used in practice, namely in the context of combining the results from multiple replicates to get a final result. Typically, a mean across three successful tests would be used. The comparison above assesses the impact of including a single test with a sigh in place of one of these. For reference, we also calculated 95% limits of agreement for comparing single replicates only. The formulae used for both types of limits are given in the Appendix.

## Results

### Participants

Lung function test results were available for 767 tests from 246 infants. The number of successful tests with and without a sigh varied for each infant. Table1 shows the number of tests for each of the five scenarios.

**Table 1:** The distribution of scenarios observed in the BIS project data. In other words, the number of acceptable MBW tests included in this study, split by the presence and location of the sigh in each test

Scenario (presence/location of sigh)	Number of tests
None	648
Washin-pre	50
Washin-post	3
Washout-pre	56
Washout-post	10
Total	767

The baseline characteristics and overall MBW outcomes (from acceptable testing cycles without sighs) of these infants are summarized in Table2.

**Table 2:** Baseline characteristics and overall MBW outcomes (from acceptable testing cycles without sighs) of the infants included in this study. For baseline characteristics, we show simple summary statistics: the count for sex and the sample mean and standard deviation for the other variables. For the outcome measurements, we show the fitted mean and standard deviation for the ‘none’ scenario (representing tests without sighs)[Table-fn tf2-1]

Characteristic (at time of testing)	Observed distribution
Sex	123 male, 123 female
Weight (kg)	4.7 (0.7)
Height (cm)	56 (2.6)
BMI (kg/m^2^)	15 (1.7)
Age (days)	43 (12)

Outcome measurement	Fitted distribution
LCI	6.8 (0.44)
CEV (L)	0.68 (0.046)
FRC (L)	0.088 (0.0072)
MR1	1.99 (0.12)
MR2	7.2 (0.89)

This choice of summary was motivated by the unbalanced replication structure of the data. A ‘simpler’ summary of the outcome measurements is only available at the expense of excluding much of the data (to get a smaller data set without replicates).

### Frequency of sighs during MBW testing cycles

As shown in Table1, about 15% (119/767) of technically acceptable tests contained a single sigh. Under a most conservative approach, these would be excluded from analyses. Furthermore, the remaining 648 tests would be evaluated against the reproducibility criteria before they would be considered further. Applying the criteria left 537 acceptable tests, across 161 individuals.

Our analysis of the impact of sighs shows that tests where a single sigh occurs in the washin-pre phase do not appreciably impact the outcome measurements (see the next section). To assess the benefit of including such tests into a study sample, we applied the reproducibility criteria to the combined set of tests (the 648 non-sighs tests together with the 50 tests with a washin-pre sigh). This gave us 591 acceptable tests, across 170 individuals. Compared to using only tests without sighs, this was a boost of 10% to the number of tests and 6% to the number of individuals included.

### The influence of sighing respirations

We fitted a linear mixed model to each outcome variable to assess the influence of sighs. The number of tests available in two of the scenarios (washin-post and washout-post) was too small to be adequately fit by the model so both were excluded from the analysis. In addition, three tests were found to have unusually extreme values for some outcome variables and substantially influenced the fit of the model: two tests had LCI > 9.5, and one had CEV > 1.4 and LCI > 9. All three had a sigh in the washin-pre phase. We excluded these three tests from our analysis based on the principles described earlier (see Methods; note that applying the reproducibility criteria leads to the exclusions of these three tests). The final model was therefore fitted on 751 tests (648 no sighs, 47 washin-pre, 56 washout-pre).

Table3 shows parameter estimates from the fitted models, with their associated 95% confidence intervals. We can see clearly different behavior for the two types of sighs under consideration. Sighs that occurred during the washin-pre phase did not discernibly influence the outcome variables: both the mean and variance were largely similar across all five outcome variables as compared to tests without sighs. In contrast, sighs occurring during the washout-prephase were associated with a small but discernible increase in the mean and variance for four of the outcome variables: LCI, CEV, MR1, and MR2. For example, the mean LCI increased by 0.36 (95% CI: 0.11–0.62), which is about 0.8 SD (the standard deviation of LCI for tests without sighs was estimated to be 0.44), and the standard deviation increased by a multiplicative factor of 2 (95% CI: 1.6–2.5).

**Table 3:** Parameter estimates for the model fit to the BIS project data. Shown are the maximum likelihood estimates (for fixed effects) or the best linear unbiased predictors (BLUPs; for random effects), and the associated 95% confidence intervals. See the Appendix for the definitions of each parameter

Parameter	LCI	CEV	FRC	MR1	MR2
Mean effects					
* μ*	6.82 (6.76, 6.88)	0.682 (0.665, 0.699)	0.0876 (0.0849, 0.0902)	1.99 (1.97, 2.01)	7.17 (7.03, 7.31)
* α*_washin-pre_	−0.135 (−0.278, 0.00813)	−0.00418 (−0.0264, 0.0181)	0.000503 (−0.00265, 0.00365)	−0.0432 (−0.0802, −0.00627)	−0.336 (−0.616, −0.0564)
* α*_washout-pre_	0.362 (0.107, 0.616)	0.0520 (0.0191, 0.0848)	0.00226 (−0.00128, 0.00581)	0.0578 (0.00224, 0.113)	0.695 (0.205, 1.19)
Variance components					
*ω*	0.296 (0.204, 0.429)	0.129 (0.115, 0.143)	0.0193 (0.0174, 0.0214)	0.113 (0.0929, 0.137)	0.849 (0.688, 1.05)
* τ*	0.186 (0.0759, 0.455)	0.0288 (0.0107, 0.0774)	0.00609 (0.00446, 0.00831)	0.0527 (0.0259, 0.107)	0.379 (0.161, 0.892)
* σ*_none_	0.437 (0.408, 0.469)	0.0455 (0.0425, 0.0487)	0.00716 (0.00669, 0.00767)	0.116 (0.108, 0.124)	0.890 (0.831, 0.954)
Ratios of residual standard deviation (compared to no sigh)					
* σ*_washin-pre_/*σ*_none_	0.823 (0.601, 1.13)	1.16 (0.710, 1.89)	0.535 (0.269, 1.06)	0.683 (0.425, 1.10)	0.694 (0.432, 1.11)
* σ*_washout-pre_/*σ*_none_	2.04 (1.64, 2.54)	2.38 (1.84, 3.09)	1.15 (0.804, 1.66)	1.57 (1.24, 1.98)	1.89 (1.51, 2.35)

**Figure 2:**
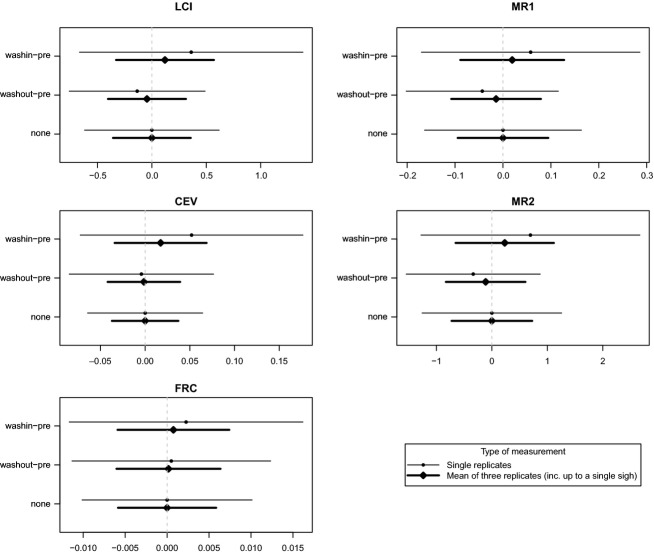
Ninety-five percent limits of agreement for the 5 MBW outcome variables, calculated using the fitted model. Comparisons are only shown for the three scenarios for which sufficient data were available to fit the model. For each scenario, two types of limits are shown, comparing different numbers and combinations of replicates (see Methods). Note that the comparisons for the ‘none’ scenario do not involve any sighs.

To quantify these effects in a more familiar measurement comparison framework, we calculated 95% limits of agreement. We did this both for the comparison of single replicate measurements only (either with or without a sigh) and the comparison of means of three replicate measurements (either all without a sigh or with one of the replicates having a sigh). The latter is the more standard scenario for these types of measurements and thus represents a more relevant comparison. The intervals are shown in Fig.2.

## Discussion

As far as we are aware this is the first study to investigate the influence of respiratory sighs on MBW measurements conducted during natural sleep in young infants. Our findings confirm that sighs are a frequent phenomenon. They indicate that sighs during the main part of the washout may influence both the magnitude and variability in MBW measurements with the associated mean increase in LCI approximating to about 0.8 standard deviations for this measurement, a magnitude that confirms the current view that these results should be excluded. On the other hand, sighs during the main part of the wash-in phase (before the last 5 breaths) were not associated with differences in MBW outcomes.

The major significance of these findings is that it is reasonable to accept data from MBW testing cycles in which a sigh occurs during the main part of the wash-in phase (after applying appropriate reproducibility criteria), particularly when the main interest is in the five outcome variables we measured. This is useful given the challenges of obtaining MBW measurements in infants during natural sleep and would result in an increase in successful tests of approximately 10% when compared to excluding them entirely.

A subtle and useful point about the inclusion of extra tests is worth mentioning. In our data, the addition of 50 tests (with a washin-pre sigh) led to an extra 54 tests considered acceptable. This perhaps counterintuitive fact is due to the new tests combining with some of the previous tests to pass the reproducibility criteria together. In other words, part of the benefit of including extra tests is they can allow the use of existing good quality data that would otherwise be discarded.

The limits of agreement analysis (Fig.2) showed the relative impact of sighs in the context of total measurement variability. In particular, there was substantial measurement variability for all of the outcome variables. The impact of even the washout-presighs, although clearly discernible, was relatively minor in comparison.

We did not have enough data to measure the effect of sighs in some parts of the testing cycle (specifically, sighs during the ‘post’ scenarios). However, this lack of data also indicates that such sighs occur rarely, and that therefore the possible benefit of their inclusion will be relatively minor.

We used a different threshold window for sighs during wash-in (last 5 breaths) than the current guidelines recommend (last 10 breaths), and similarly for sighs during washout (going beyond just the first 10 breaths). This was due to the fact that we recorded our data before the publication of these guidelines. A direct evaluation of those criteria is therefore not possible, but we can make some broad comparisons. Although our threshold for sighs during wash-in was less stringent, we nonetheless showed that the resulting impact of the sighs was negligible. This suggests that the current guidelines may be too conservative in that respect, at least for lung functions tests under conditions similar to ours. In contrast, our threshold for sighs during washout was more stringent, and we showed that these sighs have a discernible impact on the outcome. In that respect, the current guidelines might not be conservative enough.

The strengths of this study include a large, population-derived sample of infants, as well as the application of a standardized and stringent testing protocol in a single center in accordance with recent international guidelines and recommendations. The findings are likely to be relevant to studies involving MBW using SF_6_ in young infants, but it is uncertain whether they are relevant to MBW testing among older participants, or when using a different inert gas or testing device. However, sighing respirations are not usually witnessed in older subjects.

The limitations of this study include a lack of current knowledge regarding the relationship between sighs and other factors that may influence the variability in the data. These include the adequacy of equilibration of exhaled SF_6_ during the wash-in phase, the stability of the tidal breathing pattern, the stability of the end-expiratory lung volume, and the presence of mask leaks. However, current guidelines recommend the exclusion of testing cycles with sighs, independent of the relationship between sighs and other factors that may influence the variability in the data. Therefore, this limitation is unlikely to alter our estimate that the retention of data from testing cycles with sighs during the wash-in phase is associated with an approximately 10% increase in acceptable testing cycles.

The physiological basis of sighing during sleep is, at least in part, to prevent areas of lung collapse (Davis and Moscato 1994). One might therefore expect that sighs would be associated with improved ventilation homogeneity, and accordingly, a reduced CEV and LCI. In this study, however, sighs during the washout phase were associated with an increase in the CEV and LCI. This paradox may be due to an effect on MBW measurements rather than true ventilation homogeneity. Specifically, sighs during washout may be associated with release of the SF_6_ from areas of airway closure or parts of the lung where gas trapping was overcome by the sigh, but where inhomogeneity still exists. This would be associated with an increase in CEV but minimal change in FRC, as we observed. We also speculate that infants in whom sighs have a greater impact on MBW measurements may have more extensive areas of gas trapping. If genuine this phenomenon may have clinical relevance to airway diseases such as infant wheezing and cystic fibrosis where air-trapping is a common early structural and physiological manifestation of disease. It may be of interest, therefore, to investigate the relationship between the influence of sighs on MBW as a dimension of lung function measurement rather than treat sighs as a nuisance process issue only.

## Appendix

### Specification of the statistical model

We fitted the following linear mixed-effects model to each of our outcome variables:

where:

- *y*_*isr*_ is the value of the outcome variable of interest, for the *r*th replicate of scenario *s* for the *i*th individual
- *μ* is the overall mean for the no-sighs scenario
- *α*_*s*_ is a fixed effect for scenario *s* (*s* ∈ {none, washin-pre, washin-post, washout-pre, washout-post{), showing the change in overall mean compared to the no-sighs scenario (i.e., *α*_none_ = 0)
- *u*_*i*_ is a random effect to capture the variation between individuals, represented by the variance component *ω*^2^
- *c*_*is*_ is a random effect to capture any interaction between individuals and scenarios, quantified by the variance component *τ*^2^
- *e*_*isr*_ is a random effect to capture the residual variation across replicates and is allowed to vary by scenario, quantified by variance components

This model is similar to that used by Carstensen et al. (2008), but models the variation between individuals as a random effect.

### Calculation of the limits of agreement

Two types of limits of agreement were calculated. The first type compares the mean of three replicates from tests without sighs, against the mean of another three replicates from two tests without a sigh and one test with a sigh. These were calculated as follows:

where the parameters with subscript *s* relate to the specific sigh scenario under consideration, and those with subscript ‘none’ relate to the scenario with no sigh.

The second type compares single replicates only, one with a sigh and one without. These were calculated as follows:
